# Interventions to reduce postpartum stress in first-time mothers: a randomized-controlled trial

**DOI:** 10.1186/1472-6874-14-125

**Published:** 2014-10-15

**Authors:** Hibah Osman, Matilda Saliba, Monique Chaaya, Georges Naasan

**Affiliations:** Department of Family Medicine, American University of Beirut Medical Center, Beirut, Lebanon; Research Institute of McGill University Health Center, Montreal, Canada; Department of Epidemiology and Population Health, American University of Beirut, Beirut, Lebanon; Memory and Aging Center, Department of Neurology, University of California, San Francisco, USA

**Keywords:** Postpartum, Stress, Intervention studies, Hotlines, Film

## Abstract

**Background:**

The postpartum period can be a challenging time particularly for first-time mothers. This study aimed to assess two different interventions designed to reduce stress in the postpartum among first-time mothers.

**Methods:**

Healthy first-time mothers with healthy newborns were recruited from hospitals in Beirut, Lebanon after delivery. The two interventions were a 20-minute film addressing common stressors in the postpartum period and a 24-hour telephone support hotline. Participants were randomized to one of four study arms to receive either the postpartum support film, the hotline service, both interventions, or a music CD (control). Participants were interviewed at eight to twelve weeks postpartum for assessment of levels of stress as measured by the Cohen Perceived Stress Scale (PSS-10).

**Results:**

Of the 632 eligible women, 552 (88%) agreed to participate in the study. Of those, 452 (82%) completed the study. Mean PSS-10 scores of mothers who received the film alone (15.76) or the film with the hotline service (15.86) were significantly lower than that of the control group (18.93) (p-value <0.01). Among mothers who received the hotline service alone mean PSS-10 score (16.98) was also significantly lower than that of the control group (p-value <0.05).

**Conclusions:**

Both our postpartum support film and the 24-hour telephone hotline service reduced stress in the postpartum period in first-time mothers. These simple interventions can be easily implemented and could have an important impact on the mental wellbeing of new mothers.

**Trial registration:**

The trial was registered with clinicaltrials.gov (identifier # NCT00857051) on March 5, 2009.

## Background

The postpartum period is a stressful time for women, especially for primiparous mothers [1]. Besides common postpartum stressors like sleep deprivation, hormonal changes, and the demands of caring for a newborn, first-time mothers have to adapt to their new parenting role [1]. First-time mothers may feel insecure about their abilities to nurture an infant [2]. They often feel overwhelmed, exhausted, and isolated in their new roles [1]. Studies have shown that first-time mothers are at a greater risk of postpartum mental disorders than multiparous mothers [3].

Although postpartum stress has been found to directly correlate with minor postpartum psychiatric illnesses [4, 5] including postpartum depression [6–9] and anxiety [10], very few studies have evaluated interventions aiming at reducing postpartum stress. Most preventive postpartum mental health trials targeted depression as their main outcome and most targeted women at risk [11].

In Lebanon, Chaaya and colleagues [12] showed that the prevalence of postpartum depression is approximately 21%. A recent study [13] of perceived stress among postpartum women in Lebanon found that the mean stress score was high when compared to scores in other countries [7].

Interventions that reduce postpartum stress may facilitate the transition into motherhood for first-time mothers.

This study aimed to assess the impact of two simple and flexible interventions on reducing postpartum perceived stress among first-time mothers in Beirut-Lebanon: (1) a postpartum support film and (2) a 24-hour telephone hotline service. These interventions were developed because they were simple, cheap, and could be easily reproduced and adapted for implementation in different settings.

## Methods

This study was approved by the American University of Beirut Institutional Review Board in accordance to the National Institute of Health guidelines. The trial was registered with clinicaltrials.gov (identifier # NCT00857051) on March 5, 2009.

### The study participants

#### Eligibility criteria

It is estimated that 90% of all deliveries in Lebanon occur in the hospital setting [14]. All first time mothers who were delivering in hospitals in Beirut or its surrounding suburbs were eligible for inclusion in the study. They were excluded if they had any of the following characteristics: (1) had a multiple or complicated gestation, (2) had a chronic disease that required daily management (such as cardiovascular, hypertension, diabetes, or thyroid diseases) and (3) had a baby who required neonatal intensive care. Many women from neighboring countries come to deliver in Beirut and return to their home countries soon after delivery, which would make them inaccessible for a postpartum assessment. We therefore also excluded women who were expected to travel before the assessment would take place.

#### Study setting

All hospitals (26 private and one public) in Beirut and close suburbs with maternal services were contacted through a written letter and a meeting with the respective directors to seek their approval for recruiting women. In hospitals where there was an ethics board, applications were submitted for their approval. Twenty-three hospitals agreed to participate; one public (Rafic Hariri University Hospital) and 22 private (American University of Beirut Medical Center, Trad Hospital and Medical Center, Najjar Hospital, Fouad Khoury Hospital, Saint George Hospital Universtiy Medical Center, Makassed Hospital, Bahman Hospital, Sahel General Hospital, Abou Jawdeh Hospital, Al Zahraa University Hospital, Al Rassoul Al Aazam Hospital, Child and Mother Welfare Hospital, Lebanese Canadian Hospital, Saint Therese Hospital, Saint Charles Hospital, Maarbes Hospital, Lebanese Jeitawi Hospital, Beirut General Hospital, Mount Lebanon Hospital, Sacred Heart Hospital, Al Hayat Hospital, and Saint Joseph Hospital).

### The study intervention

Participants were randomized to four arms: postpartum support film, the hotline service, the postpartum support film and the hotline service, or the control group (received a CD of children’s music).

#### Postpartum support film

The intent of the film is to reduce perceived stress through modifying the cognitive appraisal of postpartum stressors. The literature shows that providing new mothers with a proactive honest reality based approach by changing maternal expectations in the postpartum period would facilitate the transition [15]. Nelson anticipated that telling mothers directly that it is normal to feel overwhelmed, uncertain, and mentally and physically fatigued in the first months postpartum and that assuring them that it is a transient and common experience can help new mothers during the transition [15, 16].

The postpartum support film was approximately 20 minutes recorded on a DVD. The film targeted both mothers and fathers and addressed the main stressors in the postpartum period as identified by a previous qualitative study [17]. These included: sleep deprivation, postpartum blues and depression, breastfeeding difficulties, return to work, postpartum sexuality and body changes. The material included presentations by two physicians and testimonials from parents. Although the film included some health related information (e.g. signs that indicate the infant is receiving adequate amounts of breast milk), the main intent was to reassure mothers that the stressors they are experiencing are common and transient. Actors representing first-time mothers were included to provide humor about the stressors.

#### Hotline service

The intent of the hotline is to provide immediate reliable answers to concerns that a mother might have. Our hypothesis was that women may be comforted by simply knowing that they had someone reliable to call should they need to regardless of whether they access the hotline or not, and that this would reduce stress. This idea was based on our clinical experience with first-time mothers [17].

The hotline was a telephone number that a mother could call at any time of the day or night to get answers to questions regarding parenting, infant care and self-care issues. The person answering the hotline was a parent or a midwife, trained according to fixed algorithms that were prepared and modified following findings from a previously published pilot hotline study [14]. Algorithms covered topics related to breastfeeding, colic, postpartum blues/depression, and normal newborn care.

### The study outcome

The main outcome of the study was perceived stress as measured by Cohen Perceived Stress Scale (PSS-10) [18]. PSS-10 has been used to assess perceived stress in a number of different populations including university students, the elderly as well as pregnant and postpartum women [13, 19–21]. It was translated into Arabic and validated in Lebanon [13].

To evaluate the efficacy of the interventions, the difference of PSS-10 means between each intervention group and the control group was measured at 8–12 weeks postpartum.

### Sample size

The mean score for the PSS-10 was found to be 18.3, with a standard deviation of 4.9 in the validation study among postpartum women in Lebanon [13]. Sample size was calculated based on the aim of reducing the PSS-10 mean by 4 points. Assuming that 50% of women in the intervention arm would watch the film, the mean for the intervention group was considered to be 16.3. Therefore, 126 women were needed in each arm with an alpha of 0.05 and a power of 90%. A total of 140 women was needed for each arm to allow for 10% loss to follow up.

### Randomization

We used a randomized controlled single-blinded design. The film, the hotline card, the film and hotline card together, or the music CD were placed in hard CD covers which were then placed in sealed opaque envelopes consecutively numbered based on a computer generated random list. All envelopes looked and felt the same. Recruiters were blinded to the content of envelopes. Each participant received the next envelop in the series when she enrolled in the study.

### Recruitment

Baseline data were collected daily over a seven-week period in March and April, 2009 by eight recruiters. Recruiters included midwives and public health graduate students who had been trained for the recruitment process. Recruiters visited their assigned hospitals at the same time every morning. At each hospital visit, the recruiter reviewed the list of deliveries in the last 24 hours and visited every woman who met the inclusion criteria in her room. Written informed consent was obtained from each woman before recruitment. After consenting the woman and providing her with the intervention envelope, a three-minute baseline interview was conducted to gather information about woman’s health, the health of her baby, pregnancy and delivery experience, as well as socio-demographic factors.

### Follow up assessment

Data collection in the postpartum period was conducted by 14 trained assessors. Most of the interviews (72%) were conducted face to face in women’s homes; the other 28% were conducted by telephone due to difficulties in reaching participants in their homes. The postpartum interview lasted between 30 to 50 minutes. In addition to the PSS-10, it included a depression scale, and anxiety scale and questions about general health, mental health, the health of the baby, and social support.

### Data analysis

The data was analyzed using the Statistical Package for the Social Sciences (SPSS) version 16.

Sample characteristics at recruitment and at assessment were presented across each arm. The differences of these characteristics among the four arms were tested using chi-squared test. Significance was considered at p-value <0.05. Sample characteristics at baseline included socio-demographic variables such as age, education level, employment and household income. Age was divided into quartiles. Education level was categorized into four groups: ‘none/primary’, ‘middle’, ‘secondary’, and ‘university’. Employment was defined as full-time or part-time. Monthly household income was categorized as less than one million, one to two million, and greater than two million Lebanese Pounds (One million Lebanese Pounds = 660 US dollars). Other indicators included Gravida (Primi/Multi), mode of delivery (Vaginal/C-Section), planned pregnancy (yes/no), and infant gender (female/male).

At follow up, sample characteristics in the postpartum were compared. Maternal variables included mother’s health problems (yes/no), breastfeeding (exclusive/mixed/formula only), and smoking (yes/no). A woman was considered a smoker if she had ever smoked in her life due to low frequencies. Infant characteristics included infant health problems (yes/no) and infant’s behavior (fussy/non-fussy). Non-fussy infants included easy infants and infants requiring medium effort. Perceived social support in the postpartum was categorized as adequate or inadequate. Marital relationship was divided into satisfactory or not. Life events were assessed by asking women if they had any major events or problems in the last year.

The impact of the interventions was evaluated using intent to treat analyses. The means of PSS-10 were compared between each intervention group and the control group using T-tests. The level of significance was set at p ≤ 0.05 for all statistical analyses.

## Results

**Figure 1:**
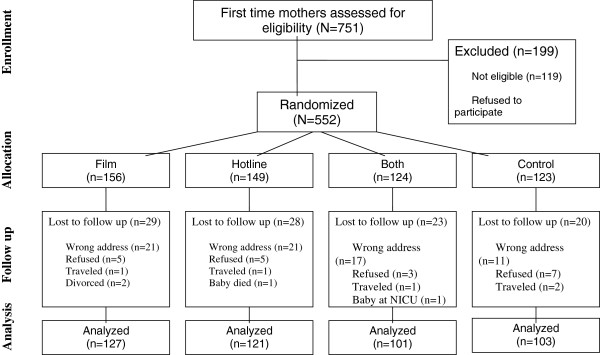
**Flow diagram of the study participants.**

Of the 751 primiparous women who were approached 119 were excluded and 80 refused to participate (see Figure 1). There were no significant differences in the socio-demographic characteristics of women who participated and those who refused based on the chi-square test at p-value < 0.05 (Table 1).

**Table 1:** **Socio-demographic characteristics of women who participated in the study**

	All	Participated (N = 552)	Refused	P-Value
	(N = 632)		(N = 80)	
Woman’s age				0.34
<= 22	24	25	17	
23–25	19	18	26	
26–29	28	28	28	
30+	29	29	29	
Woman’s education*				0.27
None/primary	5	5	12	
Middle	26	26	27	
Secondary	19	19	18	
University	50	50	42	
Mode of delivery*				0.29
Vaginal	54	53	58	
C-Section	46	47	40	
Gravida				0.18
One	89	88	93	
More than one	11	12	7	
Baby gender				0.96
Female	48	48	47	
Male	52	52	53	

*Columns do not add up due to missing values.

Of the 552 participants, 452 were assessed at 8–12 weeks postpartum and 100 were lost to follow up (see Figure 1). There were no significant differences in the socio-demographic characteristics of women who were assessed and those who were lost in follow up (see Table 2).

**Table 2:** **Socio-demographic characteristics of women who were lost in follow up as compared to those who were assessed (%)**

	Lost in follow up (N = 100)	Assessed (N = 452)	P-Value
Woman’s age			0.61
<= 22	26.0	24.8	
23–25	22.0	17.2	
26–29	24.0	28.8	
30+	28.0	29.2	
Woman’s education			0.28
None/primary	8.1	3.8	
Middle	27.3	25.4	
Secondary	19.2	19.6	
University	45.5	51.2	
Woman employment			0.34
Yes	69.7	62.1	
No	30.3	37.9	
Mode of delivery			0.63
Vaginal	51.0	53.7	
C-Section	49.0	46.3	
Gravida			0.43
One	86.0	88.8	
More than one	14.0	11.2	
Baby gender			0.89
Female	49.0	47.8	
Male	51.0	52.2	

*Columns do not add up due to missing values.

### Participant characteristics

Table 3 presents the distribution of participants at recruitment according to basic characteristics by intervention arm allocation. Overall, women’s age at delivery ranged between 15 and 50 years with a median of 27 years. Most women were educated with 50% of women having a university degree. More than one third of the women were employed (36.5%). Forty one percent of first time mothers reported a household income of less than one million Lebanese pounds. Most were primigravida (88.3%), and nearly two thirds had planned their pregnancy. Cesarean section rate was high - reaching 47% among the sample. Infant gender was divided almost equally between males (52%) and females (48%). There was no significant difference in the baseline characteristics of participants between the intervention arms.

**Table 3:** **Percent distribution of participants by baseline characteristics (at recruitment) and intervention arm**

	All arms	Film	Hotline	Both	Control	P-Value
	(N = 552)	(N = 156)	(N = 149)	(N = 124)	(N = 123)	
Age (years)						0.077
<= 22	25.0	33.8	20.1	19.5	25.4	
23–25	18.1	16.2	16.1	20.3	20.5	
26–29	27.9	20.1	32.9	33.3	26.2	
30+	29.9	29.9	30.9	26.8	27.9	
Educational level						0.360
None/primary	4.6	7.2	3.4	2.5	4.9	
Middle	25.7	25.5	26.5	27.0	23.8	
Secondary	19.5	16.3	23.8	14.8	23.0	
University	50.2	51.0	46.3	55.7	48.4	
Employed						0.878
Yes	36.5	34.0	38.1	37.9	36.1	
No	63.5	66.0	61.9	62.1	63.9	
Household income (monthly LL)*						0.370
< 1 million	41.1	41.9	39.8	38.8	43.9	
–2 million	37.1	30.2	40.6	35.7	35.7	
>2million	21.9	27.9	19.5	20.4	20.4	
Gravida						0.180
One	88.3	90.3	91.3	87.1	83.3	
More than one	11.7	9.7	8.7	12.9	16.7	
Planned pregnancy						0.307
Yes	66.5	69.0	60.4	69.9	67.2	
No	33.5	31.0	39.6	30.1	32.8	
Mode of delivery						0.392
Vaginal	53.2	59.1	50.7	50.8	51.2	
C-Section	46.8	40.9	49.3	49.2	48.8	
Baby gender						0.842
Female	48.0	45.5	50.3	49.2	47.2	
Male	52.0	54.5	49.7	50.8	52.8	

*Columns do not add up due to missing values.

Table 4 describes main health and social characteristics of first-time mothers in the postpartum period. Few women (18%) stated that they had a physical problem. Thirty five percent of women were smokers but most stated that they have stopped smoking during pregnancy.

**Table 4:** **Percent Distribution of participants by health and social characteristics at 2–3 months postpartum and intervention arm**

	All arms	Video	Hotline	Both	Control	P-Value
	(N = 452)	(N = 127)	(N = 121)	(N = 101)	(N = 103)	
Mother had physical health problem						0.594
Yes	18.4	17.5	21.5	19.8	14.7	
No	81.6	82.5	78.5	80.2	85.3	
Smoking						0.706
Never	64.8	69.1	62.8	63.6	63.1	
Ever	35.2	30.9	37.2	36.4	36.9	
Breast feeding						0.498
Exclusive	27.4	27.6	33.9	27.7	19.4	
With formula milk	39.6	40.2	38.0	36.6	43.7	
Only formula milk	33.0	32.3	28.1	35.6	36.9	
Baby had health problem						0.498
Yes	28.8	26.0	33.1	30.7	25.2	
No	71.2	74.0	66.9	69.3	74.8	
Infant’s behavior						0.578
Easy to normal	67.0	67.7	66.9	71.3	62.1	
Fussy	33.0	32.3	33.1	28.7	37.9	
Marital relationship						0.203
Satisfactory	58.3	58.7	58.3	65.3	50.5	
Unsatisfactory	41.7	41.3	41.7	34.7	49.5	
Received social support						0.20
Adequate	65.9	61.1	69.7	72.0	61.4	
Not adequate	34.1	38.9	30.3	28.0	38.6	
Major life event						0.330
Yes	16.6	15.7	17.4	11.9	21.4	
No	83.4	84.3	82.6	88.1	78.6	

Up to 29% of the infants had health problems in the first 2–3 months of lives. Only 27% were exclusively breastfed. Forty percent had breast milk complemented by formula and 33% were exclusively formula fed. Baby behavior ranged from easy to very fussy. Thirty three percent of mothers reported having a fussy or very fussy baby.

Nearly half the participants (42%) were not satisfied with their marital relationships and 39% stated that they had not received adequate social support. Seventeen percent had experienced a major life event in the last year. None of the variables described above differed significantly across the four arms.

### Effect of the intervention on postpartum perceived stress (PSS-10)

**Figure 2:**
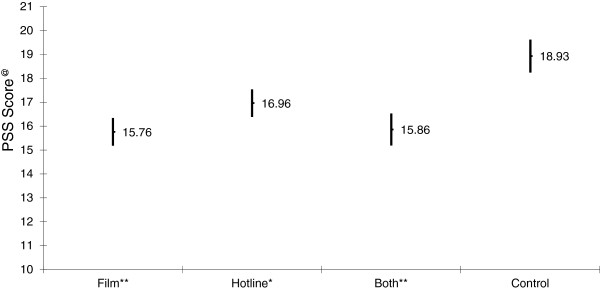
**Mean and 95% Confidence interval of PSS score by intervention arm at 2–3 months postpartum.**

Of the women who received the film as an intervention in arm 1 or 3, 61.8%reported that they had watched the film. Slightly less than one third (30.2%) of the women who were randomized to the hotline or hotline and film reported accessing the hotline. Using intent to treat analysis, the Cohen Perceived Stress Scale (PSS-10) mean of each interventional arm was compared with that of the control group (Figure 2). Results of independent T-test showed that the PSS-10 mean ± SD for first time mothers who received the postpartum support film alone (15.76 ± 6.55) or the film with the hotline service (15.86 ± 6.81) was significantly lower than that of the control group (18.93 ± 7.03) at p-value < 0.01. First time mothers who received the hotline service alone had a PSS-10 mean of 16.98 ± 6.42 which was significantly lower than that of the control group at p-value < 0.05.

Cohen Perceived Stress Scale (PSS-10) means differences between the 3 intervention arms were assessed and found not to be significant.

## Discussion

Both the film and the hotline interventions significantly reduced stress in the postpartum. Participants receiving both interventions had greater reduction in stress levels than either of the interventions alone. This difference, however, was not statistically significant.

The socio-demographic characteristics of the women in the sample were in line with characteristics of women in Beirut. Lebanese women gross enrolment rate for tertiary education is 56.3% [22] and the employment rate is 21.1% in Lebanon and 30.8% in Beirut [23]. The average of monthly household income in Beirut is 909,000 Lebanese pounds [23]. Our finding of a caesarean section rate of 46.8% approximates the rate published by local insurance companies which is 50% [14, 24].

Both the postpartum support film and the hotline service were effective in reducing early postpartum perceived stress among first-time mothers in Beirut. The impact of the film was stronger than that of the hotline although this difference was not statistically significant.

Few studies have evaluated the effect of interventions on postpartum stress. Other interventions included listening to music [25], home-based physical exercise [26], nurse or community health worker home visits [27], and postnatal support group sessions [28]. The interventions described by Dritsa et al. [26], Chen et al. [28] and Roman et al. [27] were effective in reducing perceived stress [26–28]; however, the first two studies targeted depressed women and the last study targeted women in the low socioeconomic bracket with a late assessment of perceived stress (one year postpartum). Only Tseng at al. [25] targeted women from the general population [25]; however, their intervention did not have a significant effect on postpartum perceived stress. This intervention is therefore the first effective intervention in lowering early postpartum perceived stress among first-time mothers selected from the general population.

This study has several limitations should be considered.

First, the specific aspects of the interventions that affected postpartum stress remain vague. The positive effect of the film could be derived from either the nature of the film, the messages of the film or simply just having something to watch. With regard to the hotline service, simply having access to this service, talking to someone, or having a particular problem addressed by the midwife may have impacted stress levels of the mothers. Second, the interventions reduced postpartum stress at eight to twelve weeks postpartum. Whether such an effect would last beyond that time frame remains unclear. Third, measuring stress at one point in time might have biased the results, as stress may vary from one day to next. Although the questions asked about stress on the Perceived Stress Scale addressed the stress experienced the preceding month, a mother’s immediate circumstances could affect her responses. Fourth, although we were able to demonstrate a reduction in perceived stress with both interventions, it remains unclear whether this two to three point reduction on PSS – 10 has any clinical significance. In addition, although recruiters were blinded to the intervention allocation of participants, the assessors could not be blinded since it would have been impossible to insure that participants did not mention that intervention they had received at the time of assessment. At the end of the questionnaire, the assessor asked the participant whether they had watched the film or used the hotline. This might have affected the responses of the Perceived Stressed Scale when it was not self- administered. Given that the study participants were informed at enrolment that the aim of the study was to reduce stress in the postpartum, this may have also biased their responses to the PSS-10 questions as they may have wanted to appear to respond to the intervention to please the assessors. Finally, these interventions had positive impact on new mothers in Beirut, its scope of effectiveness remains vague due to the cultural specificity of the sources of stressors.

## Conclusions

Our interventions are simple and inexpensive and can be replicated and adapted to different settings. They can be delivered through hospitals or health care centers and easily integrated into available maternal health services. Future studies should assess the impact of these interventions in different populations. The high prevalence of stress we noted in our studies of the postpartum period is concerning. Studies to understand the implications of stress in the postpartum on other aspects of mental health and the family system would be informative.

## Abbreviations

PP: Postpartum
PSS-10: Cohen perceived stress scale.
